# Cathepsin L increases invasion and migration of B16 melanoma

**DOI:** 10.1186/1475-2867-7-8

**Published:** 2007-05-08

**Authors:** Zhen Yang, James L Cox

**Affiliations:** 1 Department of Biochemistry, A.T. Still University, 800 W. Jefferson, Kirksville, Missouri, USA 63501; 2 Truman State University, Kirksville, Missouri 63501, USA

## Abstract

**Background:**

Most cancers express elevated protease levels which contribute to certain aspects of tumor behavior such as growth, metastatic spread, and angiogenesis. Elevation of the cathepsins of the cysteine protease family correlates with increased invasion of tumor cells. Cysteine proteases such as cathepsins B, H and L type participate in tumor cell invasion as extracellular proteases, yet are enzymes whose exact roles in metastasis are still being elucidated.

**Methods:**

We have examined the role of cathepsin L in highly metastatic B16F10 murine melanoma cells through genetic antisense constructs of cathepsin L. The effects of cathepsin L antisense were examined for melanoma cell proliferation, invasion, migration and adhesion.

**Results:**

Antisense expression of cathepsin L, while decreasing enzyme activity in cell lysates, did not influence cell proliferation. Cathepsin L contributed to melanoma cell invasion and also augmented melanoma cell migration. Further, we demonstrated the adhesion of cathepsin L down-regulated clones was unaltered to fibronectin, laminin, and collagen. Finally, the inhibition of melanoma cell migration via down-regulation of cathepsin L appears to be independent of cystatin C expression.

**Conclusion:**

This study shows that cathepsin L facilitates high metastatic B16 melanoma cell invasion and migration. The mechanism of migration inhibition by decreased cathepsin L is independent of cystatin C levels. Since metastasis depends upon both the invasiveness and migration of tumor cells, cathepsin L may be a therapeutic target of strong clinical interest.

## Background

When melanoma progresses to a metastatic form, it is associated with a poor prognosis. Metastatic melanoma is seldom cured with current chemotherapeutic or other regimens. New therapeutic strategies designed to deal with the metastatic spread of melanoma will require investigation of new molecular targets. Metastatic tumor cells invade host tissues through a series of steps, one or more of which requires proteolytic enzymes for invasion [1]. The cysteine proteases of the papain family, particularly cathepsins B, H and L, have been closely linked to tumor progression in multiple tumor types [2-4]. The prognosis of several major cancers has also been correlated with tumor cysteine proteinase expression in several recent studies [5,6]. Although the specific roles of cysteine proteases in metastasis remain unclear, undoubtedly these proteases participate in invasive degradation of extracellular matrix components in conjunction with other classes of proteases [7]. Modulation of tumor cell and host cell interactions by cathepsins is another important area of which little is known. Additionally, activation of other cancer cell related proteases occurs by action of cysteine proteases and contributes to tumor invasion [8].

Cathepsin L, although less well studied than cathepsin B, has been linked to tumor invasion and metastasis [9,10]. Over-expression of cathepsin L was linked to metastasis following ras transformation of NIH/3T3 cells [11]. More recently, non-metastatic melanoma cells were converted to metastatic cells by over-expression of cathepsin L [12]. Membrane association of cathepsin L in human and murine melanoma cells suggests extra-lysosomal functions of cathepsin L in metastasis [13]. Cathepsin L has additional complexity in that control of activity occurs through regulatory mechanisms which include pro-form processing of the enzyme and inhibition by proteins termed cystatins [14]. We have shown cystatin C, a potent cathepsin L inhibitor, blocks motility and invasion of melanoma cells [15,16]. The mechanism of cystatin blockade of melanoma invasion is not yet known. Cathepsin L could possibly modulate cystatin levels to influence invasion and this question prompted us to study cathepsin L further.

Although cathepsin L has been shown to contribute to melanoma cell invasion, other properties of melanoma cells such as migration, adhesion and proliferation have not been well studied. Previous studies have used synthetic cysteine protease inhibitors to study tumor-associated cathepsins which can lack specificity for cathepsin subtypes. Also, synthetic cysteine protease inhibitors can have toxic or other unwanted effects that limit conclusions in cellular studies. To circumvent problems with inhibitors, we have produced anti-sense clones to cathepsin L in B16 melanoma cells. In this study we have examined the effect of cathepsin L down-regulation on the *in vitro *behavior of highly metastatic B16F10 melanoma cells. Our results show cathepsin L makes a major contribution to melanoma cell invasion, particularly through cell migratory influences. Cell proliferation, adhesion, and cystatin C levels were not influenced by cathepsin L down-regulation.

## Results

### Initial characterization of cathepsin L antisense cathepsin L clones

**Figure 1:**
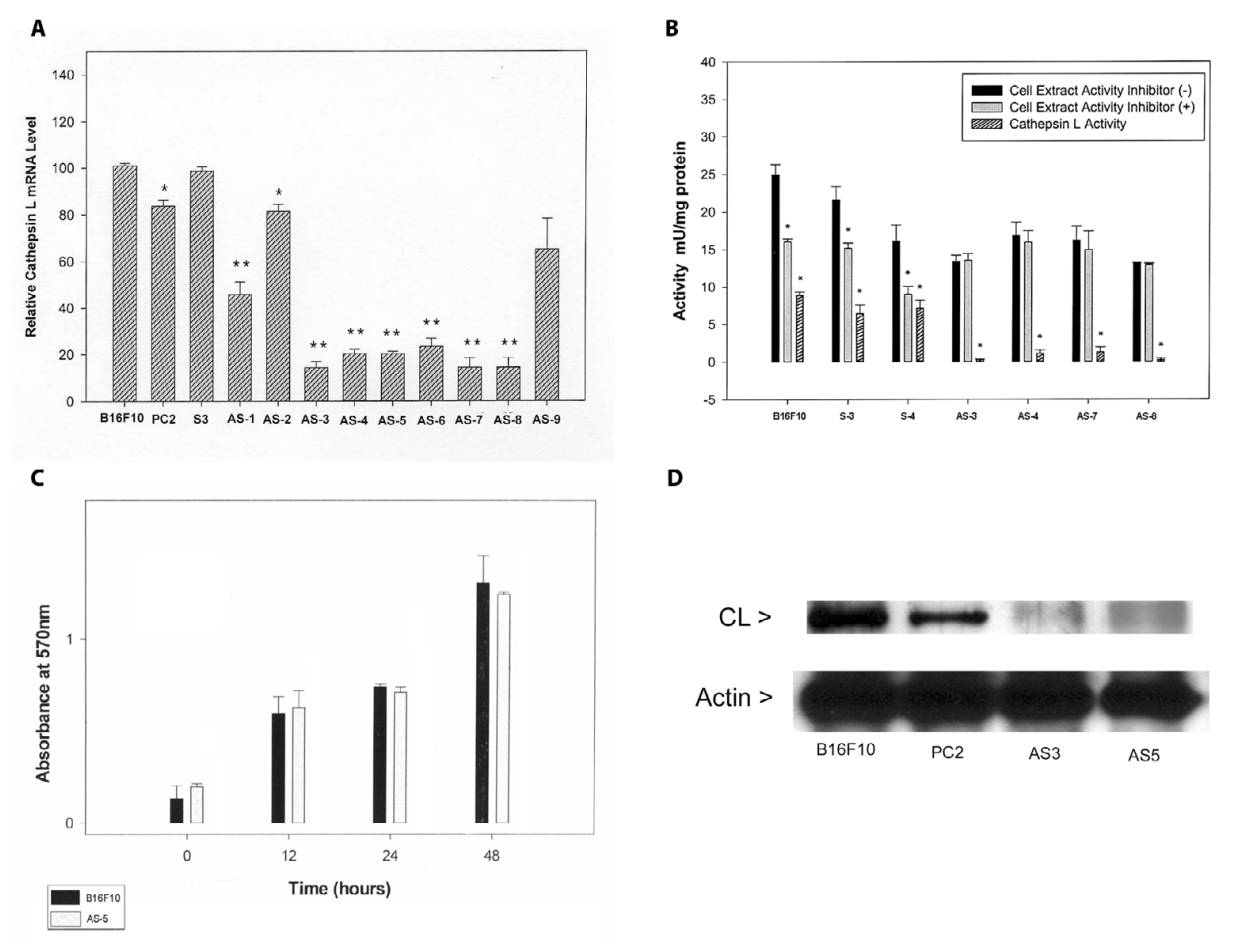
**Characterization of cathepsin L antisense clones**. **(A) **RT-PCR of cathepsin L message levels. Semi-quantitative RT-PCR was used to determine average relative cathepsin L message levels (performed in triplicate). Actin was used as an internal control and the relative product band densities were calculated based on measured pixels per band on scanned images with Image J software. Controls are B16F10 and PC2 (empty vector); AS1–9 are putative antisense clones. S3 is a sense clone. The difference between cathepsin L message levels for control and antisense clones (except AS-2 and AS-9, *P < 0.05) were significant by Student t-test (** P < 0.001). **(B) **Growth rates of cathepsin L clones. Cells were seeded in 96-well culture dishes at 1 × 10^3 ^per well. At the times indicated, MTT was added (50 ul of a 5 mg/ml solution) for incubation at 37°C for 4 hours. Media was then removed and reaction product solubilized in 100 μl DMSO for 2 hours at room temperature. Absorbances were read at A_570 _nm wavelength. Clones were run in triplicate wells in triplicate experiments. No significant difference was noted between the clones analyzed by Student t-test. **(C) **Cathepsin L activity of sense and antisense cathepsin L clones. Clones S1 and S3 are sense clones. Clones AS3, AS4, AS5, AS7, and AS8 are antisense clones. Cell extracts were prepared by lysis with incubated with Z-Phe-Arg-AMC for 30 minutes, with or without cathepsin L inhibitor in triplicate. The proteolytic activity was expressed in units (1 U = 1 μmol product per minute). ANOVA analysis showed significant differences between sense and antisense cathepsin L activities (* P < 0.01). **(D) **Western blot analysis of cathepsin L. Controls B16F10 and PC2 (pcDNA-3 vector transfected) were compared to antisense clones AS3 and AS5 for cathepsin L levels in cell extracts by Western blot analysis. Western blot detection was with ECL (Amersham) reagents.

The region of cathepsin L gene chosen for antisense construction was a 325 bp sequence contained within exon 3. We chose this sequence because it is near the 5' end of the cathepsin L message, as 5' sequences often make better antisense constructs [17]. We checked for DNA sequence similarities between the cloned cathepsin L sequence and other cysteine proteases through Genbank and found no matches. The identities of several sense and antisense plasmid constructs were confirmed by DNA sequence analysis. Cathepsin L antisense (and sense) expression plasmid pcDNA-3 constructs were transfected into highly metastatic B16F10 cells. The relative cathepsin L message level for a number of clones was determined by RT-PCR (Fig. 1A). Seven putative cathepsin L antisense clones showed significant reduction (50–80%) of cathepsin L message. Two putative clones did not (AS-2 and AS-9). In our study two clones (PC2 and AS-2) showed differences which reached P < .05 level but were much less than the clear differences for most antisense clones. We chose several clones with clearly reduced message for further study.

### Assay of cathepsin L activity

Relative cathepsin L enzyme activities were determined for several clonal cell lysates by fluorometric measurement of stopped assays. Since the protease substrate used in these assays (Z-Phe-Arg-AMC) measures primarily cysteine protease activity under the conditions used, the cathepsin L selective inhibitor Z-Phe-PheCHN2 was used in parallel assays so that cathepsin L activity could be determined by difference (Fig. 1B). Cathepsin L activities were found to be reduced by about 80% compared to control activities. We also tested purified cathepsin B with Z-Phe-PheCHN2 at the levels used in cell lysate assays and found no inhibition (data not shown). Most of the non-cathepsin L enzyme activity likely reflects cathepsin B activity that did not vary between clone types (antisense versus sense).

### Growth

We also compared the *in vitro *growth of a cathepsin L antisense clone to B16F10 parental cells under standard culture conditions (Fig. 1C). No significant difference was seen in the growth rate of an antisense clone compared to a control clone over a two-day period.

### Western blot analysis of cathepsin L

To determine if the level of cathepsin L protein correlated with relative cathepsin L message levels determined by RT-PCR, we performed a Western blot analysis of several B16 melanoma clones. The cathepsin L protein level was significantly reduced in two select cathepsin L antisense clones relative to two control clones (Fig 1D). Based on the Western blot analysis, less than half of the cathepsin L protein is present in the antisense clones tested.

### Invasion of cathepsin L antisense clones

**Figure 2:**
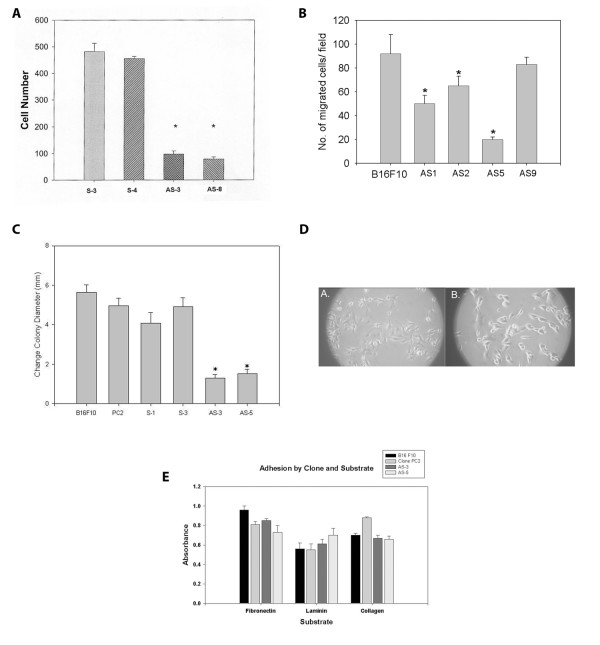
**In vitro behavior of cathepsin L antisense clones**. **(A) **Invasion assay of cathepsin L sense and antisense clones. Data represents the number of cells per filter (counted with a 40X objective) that had crossed a Matrigel barrier during 24 hours incubation at 37°C. Triplicate wells were run in triplicate. Analysis of variance (ANOVA) showed there was a significant difference between antisense clones (AS) and sense clones (S) (* P < 0.001). **(B) **Migration of cathepsin L sense and antisense clones in Boyden chamber. Data is as for invasion, but with no Matrigel barrier. Migration difference was significant by Student t test (*P < .05). (**C**) Radial migration with sedimentation chamber. Cells (2 × 10^3 ^cells per well) were plated on glass slide wells in triplicate using a cell sedimentation chamber. Chemokinetic bFGF (Sigma, 25 ng/ml) was used in these experiments. Cell colony diameters were imaged from 10X objective microscopic views. Data represent the average differences in cell colony diameter (mm) following 24-hour incubation on laminin-coated wells. Experiments were conducted in triplicate, and a significant difference was found between control and antisense clones by Student t-test analysis (*P < 0.01). (D) Morphological appearance of typical clones with light microscopy. Cells were plated on glass coverslips overnight and then images were taken using a 10 X objective. A) Control PC2 cells B) Cathepsin L antisense (AS5) cells. (E) Adhesion of cathepsin L antisense clones to extracellular matrix proteins. Plastic 96-well plates were coated with extracellular matrix proteins. Fibronectin and laminin were used to pre-coat wells with 10 μg/ml. Collagen I was pre-coated at 100 μg/ml. Cells (2 × 10^4 ^per well) were added prior to incubation for specified times. Wells were washed with PBS twice, cells fixed with 4% paraformaldehyde, and stained with crystal violet prior to reading absorbances (A_595_) of Triton X-100 (1% in PBS) lysed cells. Triplicate wells were run in triplicate and averaged. No significant difference was noted by Student's t test.

The invasion of tissue stromal and extracellular matrices is a hallmark feature of metastasis. The relative invasion of basement membrane by metastatic cells often correlates with level of proteolytic enzyme expression. A common assay used to measure relative invasiveness of cancer cells is with a Boyden chamber type assay with Matrigel coated filters. We found that the invasion of cathepsin L antisense clones was reduced by about 70% compared to control clones in a Boyden chamber invasion assay after 24 hours (Fig. 2A).

### Migration of cathepsin L antisense clones

Cell migration is another key feature underlying metastatic ability. First we looked at migration of several cathepsin L anti-sense clones with Boyden chamber assays (Fig. 2B). Cell migration of the clones paralleled the general CL message level measured (Fig. 1A). Antisense clones from the group with low CL message level (and relative migration) were focused upon for further studies. The cell migration of cathepsin L antisense clones compared to control clones was also measured with a radial migration assay (Fig. 2C) [18]. We chose this assay so that any barriers to 2d cell migration would be eliminated. In this assay cells were seeded onto laminin-coated surfaces. We found that antisense cathepsin L clones showed a 60–80% decrease in cell migration measured in a radial migration assay over 24 hours. During the course of this assay some proliferation of cells would occur. Our results on cell proliferation, however, show that observed differences in 24 hour radii between antisense cell colonies and control cell colonies are not due to proliferative differences.

### Adhesion of antisense clones

Cellular adhesion to extracellular matrices is an important determinate for cell migration. The appearance of cathepsin L antisense clones was checked for morphological differences that might account for migratory differences. After overnight culture of newly seeded cells, cathepsin L antisense clones appeared slightly more rounded (less spindle shaped) (Fig. 2D). This morphological difference may relate to reduced actin cytoskeletal remodeling expected of less migratory cells. Since adhesion of cells to extracellular matrices can impact cell migration, we tested random cathepsin L clones for adhesion to several different extracellular matrix proteins (Fig. 2E). Our results showed no significant difference in the ability of melanoma cathepsin L antisense clones to adhere to fibronectin, laminin, or collagen over a 1–2 hour period. We conclude cell adhesion of antisense clones is not a factor to account for the major differences we find in cell migration and invasion.

### Western analysis of cystatin C in cell supernatants

**Figure 3:**
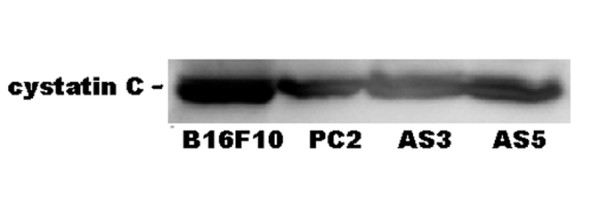
**Expression of cystatin C by cathepsin L antisense clones**. Western blot analysis of cystatin C in spent media. A representative Western blot of three independent experiments is shown.

Extracellular cystatin levels were found not to change when cathepsin L down-regulated clones were compared to control clones (Fig. 3). This result suggests cathepsin L does not regulate the level of cystatin production to influence cell migration and invasion indirectly through cystatin levels.

### Real-time PCR analysis of cystatin C

Real-time PCR was carried out for cystatin message levels in parental B16, vector control-transfected and two cathepsin L antisense clones. We found cystatin C message levels were similar in cathepsin L antisense clones compared to B16F10 or empty plasmid transfected controls (Table 1).

**Table 1:** 

**Clone**	**Ct ratio (cystatin C/actin)**
B16F10	0.94
PC2	0.92
AS3	0.97
AS5	1.00

## Discussion

In this work we have examined the *in vitro *contribution of cathepsin L to the behavior of high-metastatic B16 F10 melanoma cells. Based on earlier work in the literature, we hypothesized cathepsin L would make a major contribution to B16 melanoma invasion [19,20]. To test this hypothesis we transfected B16F10 melanoma cells with cathepsin L antisense and sense RNA expression plasmid constructs. Our cathepsin L antisense expression clones produced effective, stable, down-regulation of cathepsin L message level, protein, and enzyme activity. Both melanoma cell migration and invasion were found to be inhibited in cathepsin L antisense clones. Adhesive and proliferative differences between cathepsin L antisense and sense clones were not detected. Also, no influence of cathepsin L down-regulation on cystatin C mRNA production or secretion was found. We take these results to mean cathepsin L has direct effects on the cell motility and contributes its proteolytic action to the active invasion of melanoma cells.

Tumor invasion of host tissues requires multiple proteases, among which the catheptic cysteine proteases are typically elevated. Cathepsins B, H, and L have been shown to be elevated in many cancer types, both intracellularly as well as extracellularly [21,22]. It is known B16 melanoma cells exhibit elevated cathepsins B, H, and L levels due to increased transcription [23]. Earlier work established cathepsins B and L contribute to melanoma cell invasion *in vitro *[24].

Other properties of melanoma cells potentially influenced by cathepsins, such as adhesion, migration, and proliferation have received less study. We have addressed these parameters in this highly metastatic melanoma cell line. It would be expected that cell invasion might decrease due to reduced extracellular cathepsin L since extracellular matrix degradation by cathepsin L is known to occur. Less clear, however, is how cysteine proteases might be involved in cell migration. One way cysteine proteases might play a role in tumor cell migration is through activation of other proteases known to play a direct role in tumor cell migration [8]. Direct proof of this mechanism *in vivo *is lacking in the literature. A second clue to cathepsin involvement in tumor cell migration comes from a study of cathepsin B knockdown in glioma cells [25]. Inhibition of glioma cell migration with cathepsin B down-regulation correlated with altered cofilin phosphorylation. This is the first linkage of cathepsins and cytoskeletal changes involved in cell migration.

The relative importance of cysteine proteinases to tumor cell invasion has been addressed recently with different strategies. One study used antisense oligonucleotides to cathepsin L and demonstrated decreased migration and *in vitro *invasion of human osteosarcoma cells [26]. Differences exist, however, for the roles of cathepsin L in different cancer cell types. For example, antisense constructs to cathepsin L in human glioma cells have been shown to block invasion but not migration [19]. Intracellular expression of an antibody to cathepsin L has also been shown to block cathepsin L secretion and dramatically inhibit melanoma metastasis [27]. Secreted cathepsin L in this study was shown to be involved in both invasion and angiogenesis although migration was not measured directly.

One possible avenue towards mechanistic details concerns the use of synthetic cysteine protease inhibitors or cystatins. Most invasive tumors express elevated cysteine protease levels and at least half display decreased cysteine protease inhibitor levels relative to untransformed tissues [28]. Cysteine protease inhibitors, such as the cystatins, decrease tumor cell migration when applied exogenously, although the mechanism of migration inhibition is unclear [29]. We previously found cystatin C over-expression decreased B16 melanoma cell migration [30]. If cystatin C is over-expressed, one would expect decreased extracellular cathepsin L activity to correlate with a less migratory cell phenotype. Our cystatin C over-expression clones were found to be deficient for cystatin secretion and therefore deemed a poor model for examining extracellular cathepsin L regulation [16].

Recently, cathepsin L has been found to modulate gene transcription in NIH/3T3 cells by proteolytically activating nuclear transcription factors and suggests how protease expression might influence the expression of other genes [31]. The intriguing possibility that cathepsin L might be inducing cell migratory factors in melanoma needs to be tested with gene array analysis. Genetic support for this reasoning comes from cathepsin L knockout mice which show reduced cell migration in podocytes [32].

New therapeutic strategies that target cysteine proteases of the papain family may be able to interfere with metastatic spread of certain cancers. How central a role cysteine proteases play in cancer cell invasion depends, in part, upon the redundancy of other classes of proteolytic enzymes involved in invasion. Non-proteolytic functions of the cathepsins might also contribute to tumor cell invasion as has been found for urokinase plasminogen activator [33]. In conclusion, we have shown that cathepsin L antisense clones did not display altered cell proliferation or adhesion to different protein substrates. Cathepsin L down-regulation also does not appear to be linked to expression of the cysteine protease inhibitor cystatin C. Cathepsin L does however make a significant contribution to B16F10 melanoma cell invasion, particularly through cell migration. Cathepsin L should therefore be an anti-metastatic therapeutic target of future interest.

## Methods

### Cell culture

The highly metastatic cell line B16F10 was a gift from Dr. Isaiah Fidler (MD Anderson Cancer Center, Houston, Texas, USA). Cells were cultured in complete media: RPMI-1640 media (Sigma) supplemented with 10% fetal bovine serum (FBS, Sigma) and antibiotics (100 units/ml penicillin, 0.1 mg/ml streptomycin, 0.25 mg/ml fungizone)(Bio-Whittaker) at 5% CO_2_, 37°C in humidified cell culture chambers.

### Cathepsin L antisense DNA clones

A 325 base pair fragment corresponding to a portion of murine cathepsin L exon 3 DNA sequence was amplified from total B16F10 RNA by an RT-PCR reaction [34]. The primers used for amplification of cathepsin L exon 3 DNA sequence were left primer (5'-AGTCCACCGCACAGAAGACTGTA-3') and right primer (5'-CCGGTCTTAAGGAACATCTGTC-3'). The PCR-amplified cathepsin L DNA fragment was cloned as a blunt-ended fragment into the Sma I site of pcDNA-3 plasmid (Invitrogen) in both sense and antisense directions. Cloned cathepsin L DNA fragments were confirmed by DNA sequence analysis carried out at the University of Chicago Cancer Center. Plasmid DNA from vector, sense, and antisense clones were purified and transfected into B16F10 cells by the calcium phosphate method [35]. After 2–3 weeks growth in complete media plus Geneticin (1 mg/ml (Sigma)), well separated cell colonies were retrieved from cloning rings following brief trypsin treatment for further analysis. Quantitation of relative cathepsin L mRNA levels in selected clones was by PCR amplification of reverse transcription products. Total RNA was isolated with Tri-Reagent (Sigma) and treated with DNase I (1 unit/μg RNA) for 20 minutes prior to reverse transcription. Reverse transcription of 1 μg of total RNA was carried out with an enhanced AMV reverse transcriptase (Sigma) reaction with right cathepsin L primer (100 ng/reaction). Exon 3 of murine cathepsin L was then amplified for 30 cycles from 5 μl reverse transcription reaction product. An internal control of β-actin (0.5 μl of an actin RT-PCR reaction prepared from 1 μg B16 F10 melanoma RNA) was included in the PCR reaction to normalize the amount of cathepsin L specific products. Mouse actin primers used were AM1 (5'-ATGGGTCAGAAGGACTCCTAT-3') and AM2 (5'-AAGGTCTCAAACATGATCTGGG-3') [36]. Digital photos of amplified bands on agarose gels were scanned with a CCD camera and quantified with Scion Image Beta 4.0.2 program. The ratio of cathepsin L PCR product to actin PCR product in terms of pixels per band was calculated for each clone.

### Assay of cathepsin L activity

An enzyme assay for cathepsin L activity was used with slight modification from Kirshke et al. [7]. A stopped assay for cysteine protease activity (30 minute incubation) was carried out with Z-Phe-Arg-AMC (14 μM) (Enzyme Systems, Inc.) as substrate for fluorometric measurements. In these assays, one unit of enzyme activity equals 1 μmole of substrate hydrolyzed per minute. The specific cathepsin L inhibitor Z-Phe-Phe-N2 (Enzyme Systems, Inc.) was used at 4 μM in some assays as 0.4 μM inhibitor failed to totally inhibit cathepsin L in our assays. Cathepsin B activity was not inhibited by 4 μM inhibitor concentration *in vitro *(data not shown). Protein assays were carried out with a modified Lowry assay [37].

### Invasion and migration assays

Melanoma cell invasion was measured with modified Boyden chambers (Nucleopore) as described previously [30]. Briefly, polycarbonate filters with 8 μm pores (Nucleopore) were etched with 10% acetic acid solution overnight at 4°C. Membranes were then coated with gelatin solution (0.1%) for 4 hours at room temperature. Filters were then washed twice in PBS and air-dried in a laminar flow hood. Filters were next coated with Matrigel (BD Biosciences) (65 μg/filter) and dried before use. Cell growth media plus basic fibroblast growth factor (bFGF) (25 ng/ml) filled the lower chamber and the top chamber received 200 ul media plus 2 × 10^4 ^cells. After 24 hours at 37°C, filters were removed and cells on the bottom surface were fixed for 10 minutes in methanol. Cells were permeabilized for 2 minutes in 0.1% Triton X-100-PBS solution before staining in Harris hematoxylin solution (Sigma) for 10 minutes. After a 2-minute rinse in tap water, filters were mounted directly on glass slides for cell counting with an inverted microscope using a 40X objective. A migration assay was also performed by this same method with filters pre-coated with collagen I (Sigma) at 10 μg/ml at 4°C overnight, but no Matrigel coating. Migration of cells was for 10 hours in complete media with 25 ng/ml bFGF in the lower well.

### Radial migration assay

Melanoma cell migration was measured in a radial migration assay [18]. Ten well slides and sedimentation manifold were obtained from CSM Inc, Phoenix, AZ. Wells were coated with laminin (10 μg/ml in phosphate buffered saline (PBS) at 100 μl per well) for 1 hour at 37°C. The wells were then washed twice with PBS and air-dried. Cells were seeded through the sedimentation manifold at 2 × 10^3 ^cells per well onto glass slides containing complete cell growth media plus basic fibroblast growth factor (bFGF)(Sigma) at 25 ng/ml. All migration and invasion assays include bFGF as a chemokinetic factor to maximize cell migration rates. Incubation of the seeded cells for 4 hours allowed the cells to establish a 1 mm diameter colony of attached cells per well. An initial image was taken of the well field and then again after 24 hours to permit cell migration. Migration results are the change in the radius of the cell population over a 24-hour period. In this assay, cathepsin L antisense clones were always run on the same slides as sense clones to minimize variability. Measurements were taken with a 10X objective on an inverted microscope (Lomo Invertoscope) and digitized with a camera (Senetech 620). Image analysis was performed with Image J software.

### Cell adhesion assay

Fibronectin (Sigma) and laminin (Sigma) were diluted in PBS to 10 μg/ml, and 100 μl solutions were used to coat 96 well plates for 1 hour at 37°C. Excess solution was removed from wells followed by a wash with PBS. Wells were used immediately for adhesion assays. Collagen I (100 μg/ml in PBS) (Upstate) was used to coat wells under the same conditions. Cells were removed from near confluent dishes with 0.02% trypsin, and the trypsin was neutralized with an equal volume of complete media. Cells were then diluted in serum-free DMEM plus 0.1% BSA to a final concentration of 2 × 10^5 ^cells per ml. Suspended cells (2 × 10^4^) were added to each well for either 30 or 60 minutes incubation at 37°C (120–240 minutes for collagen I) before unattached cells were removed by washing twice with PBS. Cells were then fixed with 2% paraformaldehyde in PBS for 10 minutes at room temperature. After washing the wells with PBS, the cells were stained with crystal violet (0.1%) for 20 minutes at room temperature. Wells were then washed 3X with PBS and the cells lysed in PBS containing 1% Triton X-100 before well absorbances were read at A_595_.

### Western blots

Cellular protein was extracted from near confluent 35 mm culture dishes with lysis buffer (20 mM Tris, pH 7.5, 150 mM NaCl, 1 mM MgCl_2_, 2 mM EGTA, 10% glycerol, 0.15% SDS, 1% deoxycholate, 1% Triton X-100, 1% anti-protease cocktail (Sigma)). When cell lysates were analyzed, 50 μg total protein was loaded per sample. Cathepsin L mouse monoclonal IgG (1:1000) (Alexis) and actin rabbit polyclonal IgG (1:1000) (Santa Cruz) were used for Westerns. Immunoreactive bands were detected with horse radish peroxidase (HRP) linked anti-rabbit IgG (Santa Cruz Biotechnology) by enhanced chemiluminescence (ECL, Amersham Life Sciences). For cystatin C analysis in culture supernatants a rabbit polyclonal anti-cystatin C (1:1000) (Upstate) was used. Several melanoma cell clones were grown to near confluence and then switched to serum-free DMEM media plus 25 mM HEPES buffer for 24 hours. Spent media was then dialyzed against 1 mM HEPES, pH 8 overnight before lyophilization of the samples 10 fold. The loaded sample volumes were normalized to lysed cell protein prior to Western blot analysis.

### Quantitation of cystatin mRNA expression

The level of cystatin C mRNA expression was determined by quantitative real-time PCR analysis. Total RNA was first isolated by Tri-reagent (Sigma) and further purified over RNeasy minicolumns following DNase treatment as per manufacturer's instructions (Qiagen). Primer sets for murine cystatin C were obtained from Superarray and used for reverse transcription reactions (1 μg total RNA/reaction) with Reaction Ready first-strand cDNA synthesis kit (Superarray). Real-time quantitation was carried out for each mRNA relative to actin message with a SYBR green assay on an iCycler detection PCR system (Bio-rad). A dilution of 1:100 of cDNA was used for actin PCR template. We expressed the cystatin message level as a ratio of Ct values for cystatin relative to control actin Ct values. The Ct values were averages from triplicate PCR reactions for each sample.

## Abbreviations

bp, base pairs; BSA, bovine serum albumin; CL, cathepsin L; DMSO, dimethylsulfoxide; ECL, enhanced chemiluminescence; MTT, 3-(4,5-dimethylthiazol-2-yl)-2,5-2H-tetrazolium bromide; PBS, phosphate buffered saline, calcium free.

## Competing interests

The author(s) declare that they have no competing interests.

## Authors' contributions

ZY did the cathepsin cloning and expression work as well as enzyme assays. JC performed *in vitro *analysis of clones and Western blot analysis. Both authors read the final version of the manuscript.
